# Visual investigation of swelling and migration behavior of bentonite and kaolinite clays at elevated temperature using micromodels

**DOI:** 10.1038/s41598-025-01785-7

**Published:** 2025-05-14

**Authors:** Farshad Mostakhdeminhosseini, Yousef Rafiei, Mohammad Sharifi

**Affiliations:** Department of Petroleum Engineering, Amirkabir University of Technology, Tehran, Iran

**Keywords:** Low salinity waterflooding, Clay swelling, Clay migration, Bentonite, Kaolinite, Glass micromodel, Fossil fuels, Mineralogy, Energy infrastructure

## Abstract

Low-salinity waterflooding (LSWF) is an effective enhanced oil recovery (EOR) method, where injecting low-salinity brine disturbs the reservoir’s chemical balance to mobilize residual oil. However, clay minerals, abundant in sandstone reservoirs, pose challenges due to their tendency to swell and migrate, leading to permeability reduction and potential formation damage. While the impact of LSWF on clay-related damage is well studied, the role of temperature in exacerbating these effects remains insufficiently explored. This study investigates the effect of temperature on clay swelling and migration using a microfluidic oven and micromodels coated with bentonite and kaolinite. A series of injection tests were conducted under ambient and elevated temperatures, considering the influence of different cation types in the porous media. Image processing techniques were used to assess porosity, effective porosity, and permeability variations. The results indicate that temperature does not significantly affect clay swelling. However, higher temperatures greatly enhance clay migration for both bentonite and kaolinite, leading to severe pore throat clogging, an effect not observed at ambient temperature. These findings highlight the critical role of temperature in LSWF and its potential to exacerbate formation damage, emphasizing the need for careful reservoir management in high-temperature conditions.

## Introduction

Formation damage can occur during different stages such as drilling, workover, production, and stimulation due to the invasion of foreign fluids which disturbs the balance between formation and native fluids^1,2^. Formation damage is a function of different parameters such as pressure, temperature, capillary pressure, wettability, particle and pore size distribution, fine migration, and forces related to detachment and retention of fines, flowrate, and water salinity^3–5^. The presence of indigenous clays in most of the sandstone formations as a part of the matrix, coating on pore walls and inside of the pores is proven^6^. These clays can play a significant part in permeability reduction as they can swell and migrate, a phenomenon observed in various soil stabilization studies, including the use of ZnO tetrapods to enhance soil strength and mitigate permeability loss^7^. Due to drilling, completion, and workover operations, most of these clay-related damages are seen in near-wellbore areas. By injection of foreign fluids formation due to clays can be induced far from the wellbore and in the reservoir^8^. Most sandstone clay content consists of non-swelling clays such as kaolinite and illite, labeled as migratory or dispersible clays, making clay migration the main concern in sandstone formations. However, swelling clays can also migrate and induce even more severe formation damage^9^. The type of clay mineral and its cation exchange capacity (CEC) significantly influence LSWF performance and its induced formation damage, for example clays with high CEC, such as montmorillonite, are more likely to swell and migrate in response to salinity changes^10^. Permeability reduction due to disruption of the chemical balance of the clay-bearing formations by the introduction of low-salinity fluids is well known but the effects of disturbance in thermal balance should be further investigated^11^. It is well known that to initiate the swelling and migration-related damages in clay-containing rocks, the salinity of the formation water should be decreased below the critical salt concentration or CSC which is a function of the cation type present in the water^12^. For example, the presence of $\:{K}^{+}$ cation can lower the CSC compared to $\:{Na}^{+}$ also divalent cations can further decrease the CSC value which is translated to delaying the damage initiation at much lower concentrations^13^. Divalent cations (Ca^2+^, Mg^2+^) play a crucial role in clay swelling and stability^14^. The interlayer cations in clay minerals can be exchanged with cations from the surrounding brine. This exchange significantly affects the swelling capacity of the clay. For instance, monovalent cations like Na⁺ and Li⁺ typically lead to greater swelling compared to divalent cations like Ca²⁺, which can stabilize the clay structure. The hydration energy of these cations plays a crucial role in determining the extent of swelling, with higher hydration energy correlating with increased swelling^15^. Their presence can either stabilize or destabilize clays depending on their concentration and interaction with other ions^16^. Another damaging mechanism that can cause clay migration is the dominance of the hydrodynamic and physicochemical repulsion forces over the other attraction forces that finally result in the detachment of the clay particles from the pore walls which can be explained using DLVO theory^17^. The surface charge of clay particles can be influenced by the ionic composition of the injected water. In LSWF, the salinity of the injected water is reduced, which can alter the charge distribution on clay surfaces. This change can lead to a decrease in the electrostatic repulsion between clay particles, promoting aggregation and migration^18^. Cation exchange capacity (CEC) is a critical parameter that determines how clay interacts with ions in the solution. The presence of divalent cations (e.g., Ca²⁺, Mg²⁺) can enhance the stability of clay suspensions by neutralizing negative charges, while monovalent cations (e.g., Na⁺) may lead to increased dispersion^19,20^. The pH of the injected water affects the surface charge of clay particles. At low pH, the surface charge may become less negative, reducing electrostatic repulsion and promoting aggregation. Conversely, at higher pH levels, the negative charge increases, enhancing repulsion and stability^21,22^. Hydrodynamic forces arise from the movement of fluids and significantly impact the migration of clay particles during LSWF. The flow of injected water generates shear stress on clay particles, which can lead to their mobilization. The magnitude of shear stress is influenced by the flow rate and viscosity of the fluid^23,24^. Smaller clay particles are more susceptible to being carried away by fluid flow due to their lower inertia, while larger particles may settle more quickly. The geometry of the pore spaces in the reservoir affects how clay particles migrate. In confined spaces, clay particles may interact with the walls of the pores, which can either enhance or inhibit their movement^25^. The migration of clay particles during LSWF is governed by the combined effects of electrostatic and hydrodynamic forces. The balance between attractive electrostatic forces (which promote aggregation) and hydrodynamic forces (which promote dispersion) determines the stability of clay suspensions. When hydrodynamic forces dominate, clay particles remain dispersed; when electrostatic attractions are stronger, particles may aggregate and settle^26,27^. The migration of clay particles can be viewed as a result of the interplay between these forces. In a flowing system, hydrodynamic forces may initially mobilize the particles, while electrostatic interactions can influence their subsequent behavior, such as aggregation or further dispersion^28^.

Depending on the type of clay present in the formation, swelling, and clay migration can cause permeability reduction meaning formation damage.

Due to the rising worldwide demand for energy secondary and tertiary recovery or enhanced oil recovery mechanisms are put into work and the most common approach due to its inexpensiveness and availability is waterflooding^29,30^. LSWF involves injecting water with lower salinity and altered ionic composition compared to the formation brine to improve oil recovery^31^. Several mechanisms were reported to be involved in LSWF such as wettability alteration^32^, osmosis^33^, mineral dissolution^34^, fine migration and the resulting fluid flow diversion^35^, expansion of electrical double layer^36^, ion exchange, etc^37^. The effectiveness of LSWF is attributed to the rapid onset of the mechanisms involved, such as wettability alteration^38^. The mixing of low salinity water with formation water can also result in the precipitation of inorganic scales, such as calcium carbonate (CaCO_3_) and calcium sulfate (CaSO_4_), which can clog pore spaces and reduce permeability^39,40^.

The presence of clay minerals is often considered a necessary condition for the positive effects of LSWF. Research indicates that clays can enhance oil recovery by facilitating fines migration, which improves the flow of water through the reservoir and improving sweep efficiency^41^. While clays can enhance oil recovery, they can also pose challenges. The interaction of clay with low salinity water can induce swelling and migration of clay particles, obstructing flow paths in the reservoir. This phenomenon is particularly pronounced in mixed-wet systems, where the dynamics of two-phase flow are significantly influenced by the presence of clay^42^. The electrokinetic interactions between clay particles and the ionic species in the injected water can lead to changes in the stability of emulsions and the overall flow behavior of the fluids in the reservoir also the presence of clay can enhance the formation of asphaltene-clay complexes, which can further complicate the recovery process^43^. Excessive fines migration can lead to pore throat plugging, reducing overall permeability and hindering fluid flow^44^. During low salinity water flooding due to the fine migration especially clays, pores would be blocked and injected water would be diverted to the unswept areas which helps the sweep efficiency to be improved^45^. Water permeability will be also reduced which helps improve oil recovery^46^. Although the fine migration can enhance oil recovery, clogging the pores, especially by clays will also reduce the absolute permeability of the reservoir and induce formation damage which strongly affects the injectivity of water^28^. Kaolinite was found to be the main cause of formation damage due to its migratory nature^47,48^. Another type of damaging clays that present swelling behavior and also can migrate is bentonite which has a high swelling capacity and is abundant in oil reservoirs. It can swell up to many times its size by adsorption of water into its interlayer space due to lowering the injected water salinity and the magnitude of the swelling is controlled by the type of its exchangeable cation and formation water^49^.

By reducing the formation water salinity below the CSC value, the electrostatic attractive forces between the surface of the rock and clays will weaken, causing fine detachment and migration and also by osmotic swelling mechanism clays can swell and cause formation damage^50^. Fine migration and clays clogging the pore throats also are problematic in geothermal fields and water disposal wells^51^. For example, in the study conducted by Wang et al. which the effect of fine migration resulted from mineral dissolution at elevated temperatures in water disposal applications was investigated, it was reported to be less problematic in elevated temperatures as mineral dissolution in those temperatures helps the pores to open which limits the permeability impairment but the fact that temperature-dependent zeta potential will intensify the fine migration as temperature increases were also stated^52^. Fine detachment from the surface is a result of repulsive forces exceeding the attractive forces and loss of the balance between these forces which is explained by DLVO theory and its reliability on the fluid characteristics (such as pH, salinity, composition) and temperature^53^.

It has been well studied and investigated that fine migration is a strong function of pH and brine salinity due to their effect on the attractive forces between the pore surface and fines. Fine migration can also be seen during steam injection; therefore, it would be reasonable to study the effect of temperature on fine migration. In the case of fine stability calculations, it is safe to say that temperature plays a critical role^54^. There’s evidence that clay-related formation damage is linked to elevated temperature^55,56^.

Results of the coreflooding tests conducted at elevated temperatures (up to 150 °C) using clay-containing sandstone core plugs showed that the double-layer repulsion force had the strongest impact on formation damage due to the temperature change^57^. In the experiments conducted by Schembre et al., it was found that fine migration is a strong function of temperature and pH. They also found out that as pH and temperature increase the zeta potential becomes more negative which means that repulsive forces between clay particles and the rock’s surface will strengthen even in temperatures marginally higher than the ambient^58^. In the study conducted by Cheng and Milsch, the negative effect of the rising temperature and salinity reduction was proved on illite-bearing sandstone’s permeability^59^. In the study done by Musharova et al., fresh water was injected into the core plugs saturated with high salinity water in temperatures of 23 °C, 93 °C and 150 °C, it was found that the rate of permeability reduction was steeper in elevated temperatures but at the end magnitude of permeability reduction was the same for all the temperatures^57^.

Different studies were conducted to investigate the effect of temperature on swelling behavior and the results are contradictory. At elevated temperatures, a reduction in swelling pressure has been attributed to several mechanisms, including the loss of hydrating water, movement of water from micropores to macropores, and changes in the diffuse double layer thickness^60^. Conversely, some studies report an increase in swelling pressure with temperature, which can be explained by the effects of thermal energy on pore water pressure, hydration pressure, and osmotic pressure^61^. Overall, the temperature dependence of swelling pressure in bentonite is complex and influenced by structural and environmental factors, necessitating further research to fully understand these interactions^62^. The swelling pressure of bentonite underwent investigation in different temperatures and was reported to increase as temperature elevated^63^.

There are a lot of non-thermal ways to mitigate clay-related formation damage such as: using clay stabilizers^64,65^, clay inhibitors^66^, nanoparticles^67^, and divalent cations in the injected water but it was reported that thermal stabilization of expansive clays can be another solution^68^.

The permeability of rocks, particularly those containing clay, is a critical factor in various geological and engineering applications, including petroleum reservoir management, groundwater flow, and the thermal performance of underground structures. Recent studies have highlighted the complex interplay between temperature and the permeability of clay-rich soils and rocks^69–71^.

In recent years, new approaches for studying pore-scale phenomena have been invented, making the investigation of damaging and recovery mechanisms easier and clearer. The microfluidic device is one of those inventions that help us researchers visually study and understand the mechanisms that take place inside the porous media^72,73^. Glass micromodels besides the advantage of transparency also can be used to represent sandstone rocks if they are clay coated^74,75^. Glass micromodels have been used to study low salinity waterflooding mechanisms recovery mechanisms^76–79^ also the formation damage induced due to injection of low-salinity water was undergone investigation using glass micromodels too^80–82^. Nearly all of the investigations conducted to study the effect of temperature on damaging mechanisms related to clay were done using coreflooding apparatus which was stated before as a few examples^52,54,57,83^.

Although the coreflooding approach is more precise and representative of the real conditions of the field, micromodels due to their visible nature can offer advantages that coreflooding as a black box cannot. The aforementioned micromodel studies and nearly all of the micromodel investigations were conducted at room temperature which does not answer the question that has been raised here and it is the objective of this study which is the effect of the temperature on the clay-related formation damage. Here we used glass micromodels which are coated with different types of migratory and swelling clays such as kaolinite and bentonite and placed them in a microfluidic oven which was designed especially for this study to investigate the effect of elevated temperature on clay swelling and migration separately and in combination with each which to the best of our knowledge was never done before.

## Materials and methods

In the present study, an experimental procedure was designed to investigate the effect of temperature on the severity of clay-related formation damage such as swelling and migration in a low-salinity water flooding scenario. Glass micromodels coated with kaolinite and bentonite were implemented to mimic the sandstone porous media as much as possible. For the heating purpose of the micromodel, a microfluidic oven was used which was designed by the Poreflowtech Co. in Iran, that not only provides the temperature conditions but also is visible which helps images to be taken from the micromodel without the need to bring it out from oven and consequently retaining the temperature conditions. To reach a stable condition, high salinity brines with different compositions were injected into the micromodels, and after reaching stability fresh water was injected to induce formation damage. In certain time steps pictures were taken from the micromodel in order to obtain desired results. All of the tests were conducted both at ambient temperature and 70 °C.

### Micromodel

Glass-type micromodels were prepared for experimentation. A three-layered pattern was chosen to be etched on the glass surface in order to represent the physical properties of porous media. Micromodels were costumed using the following steps:

- Pattern was designed using CorelDraw software.
- Two glass plates with length of 16 cm, width of 8 cm and thickness of 6 mm were cut.
- The designed pattern was etched on one of the plates using the laser.
- The etched plate was scraped with a metal brush and placed in the ultrasonic bath for 10 min to be free of any glass splinters.
- Inlet and outlet paths were drilled at both ends of the etched plate.
- Using chemicals, both plates were washed.
- Two plates were put on top of each other and placed in a furnace to reach 700 °C to make sure of complete fusing.
- Inlet and outlet needles were inserted using glue.

**Fig. 1:**
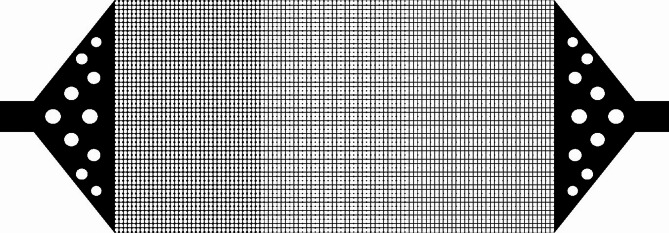
Micromodel pattern used in this study generated using CorelDraw (v24.4.0.636; Corel Corporation; https://www.coreldraw.com/en/).

The designed pattern for micromodel which is a network of pores and throats is presented in Fig. 1.

The pattern consists of three different homogenous layers with different pore sizes and the same throat size which are put together vertically. From left to right, the first layer has a pore diameter of 0.45 mm, in the second layer or the middle one, the pore size will decrease to 0.3 and in the third layer pore size will further decrease to 0.2 mm. The throat width across the whole pattern was set to 0.1 mm. Other parameters of the pattern are presented in Table 1.

**Table 1:** Micromodel properties.

Distance between the center of two adjacent pores	1 mm
Length of pattern’s porous media	102.9 mm
Width of pattern’s porous media	54.45 mm
Etching depth	0.285 mm
Pore volume	0.44 ml

### Clays and clay coating

Two types of clays were selected, kaolinite as the migration agent and bentonite as both swelling and migration agent. The kaolinite and bentonite powders were obtained from Dr.Mojalali Co. in laboratory grade. Each test will begin with coating the micromodel with clay. Clay suspension was prepared and injected into the micromodel for several pore volumes then the micromodel filled with clay suspension was placed in an oven for 1 h at 140 °C. The coating procedure is as follows:

- Preparation of 15,000 ppm NaCl solution by adding 0.3 g NaCl salt to 20 ml of distilled water. This solution is used for making clay suspension which helps prevent clay from swelling and inhibiting plugging of the micromodel and also ensures clay attachment to the glass surface.
- Making 20 wt% clay suspension by adding 1.25 g of either kaolinite or bentonite powder to the 5 ml of NaCl brine.
- Injection of 10 ml of NaCl brine into micromodel which helps the clay suspension injection easier and uniform.
- Injecting 3 ml of clay suspension into the micromodel.
- Placing clay-filled micromodel in oven with 140 °C for 1 h.
- Water will be evaporated and clays will attach to the pore and throat surface.

To ensure the reliability of the obtained results, before conducting tests, the porosity of the clay-coated micromodel saturated with high salinity brine as formation water was calculated, and it was made sure that it was relatively similar to the other tests. If it wasn’t, the micromodel again underwent the clay coating procedure to ensure that the initial porosity was similar to the other tests.

### Fluids

As was stated before in the introduction section, type of the cations present in the water can affect the severity of the induced formation damage during injection of low-salinity water. In order to investigate the effect of temperature on swelling and migration of clays in different damage magnitudes, a variety of salts with monovalent and divalent cations such as NaCl, KCl and CaCl_2_ were chosen to form 5 different high salinity fluids which will be used as formation water. By adding a certain amount of salts to distilled water, synthetic formation water with a salinity of 60,000 ppm was prepared. The composition of mentioned formation waters is presented in Table 2.

**Table 2:** Composition of formation waters used in this study.

No.	Composition	Salinity (g/L)
1	NaCl	60
2	KCl	60
3	CaCl_2_	60
4	NaCl + CaCl_2_	30 + 30
5	KCl + CaCl_2_	30 + 30

Distilled water was selected as the low-salinity water for flooding in order to induce the most severe clay swelling and migration.

### Experimental setup

**Fig. 2:**
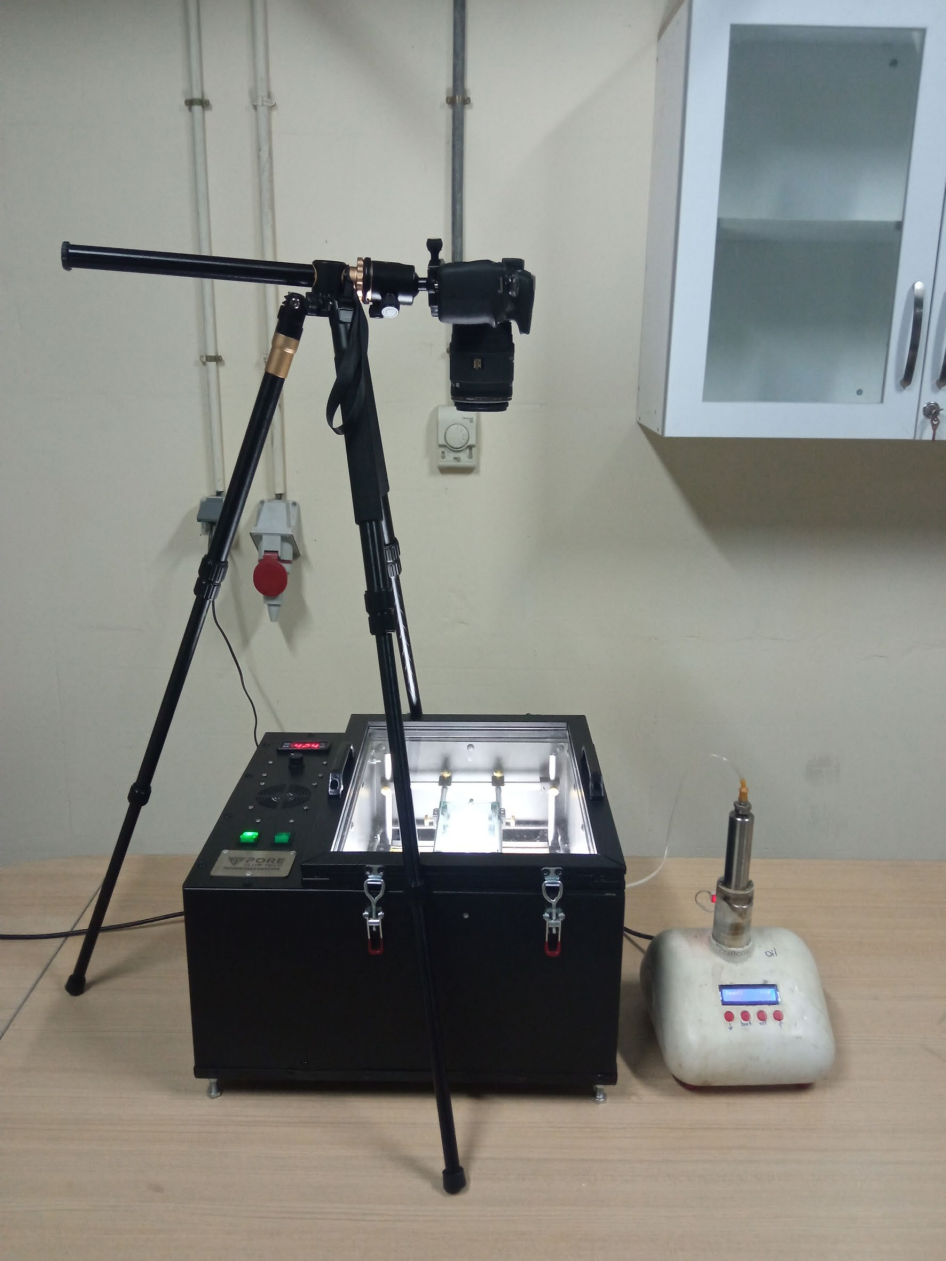
Experimental setup used in this study.

The setup used in this study consists of a high-resolution DSLR camera with a macro lens for taking pictures at short distances, a precise syringe pump with the ability to inject fluids at ultra-low flowrates, a computer system for controlling camera and picture storage and a microfluidic oven which is visible and the micromodel can be placed in it with embedded lighting system and temperature controller for stabilizing micromodels temperature. A picture of the setup is presented in Fig. 2.

### Experimental procedure

As can be seen from Fig. 2, using the syringe pump distilled water will be injected into the clay-coated micromodel which is already saturated with the desired high salinity formation water to initiate clay swelling and migration at the determined temperature. Using the high-resolution camera images were taken from a micromodel at the start of each test right before low salinity water injection and with 1 h time steps during injection of distilled water. These images were analyzed using MATLAB’s Image Processing Toolbox in order to obtain, the porosity, effective porosity and permeability of the micromodel. Detailed procedure of experimental procedure is explained below:

- At first, the micromodel was clay-coated according to the steps mentioned before.
- Clay-coated micromodel was placed in the microfluidic oven at the desired temperature.
- Desired formation water was injected into the micromodel and 2 h was given to the system so that the clays stabilize in the salinity and temperature conditions.
- The first picture was taken from the micromodel as the initial state of the clays.
- Distilled water was injected into the micromodel for 12 h at the rate of 1 PV/hr (0.44 ml/hr) and every hour pictures were taken from the micromodel.
- At last after the test, the micromodel was washed using distilled water at a high rate to be free of any clay.

This procedure was repeated for two types of clays (Kaolinite and Bentonite), five types of formation water that were mentioned before and two temperatures (25 °C and 70 °C) which sums up the total number of tests to 20 different tests. The reason for selecting 70 °C as the higher testing temperature is that it closely resembles the reservoir temperature of some of the oil fields in Iran^84^. The injection flow rate of distilled water was selected to be 0.44 ml/hr because, by this flow rate, the velocity of the injection would be around 4 ft/day which is near to the common velocity of real field waterflooding operations.

### Image processing

At first, the taken images from the micromodel will be edited using Lightroom software to be cropped and retouched in case of contrast to make the processing procedure more precise. Then the edited pictures will be masked using CorelDraw software in order to distinguish between pore space and the matrix blocks which both are colored white in non-masked pictures. A black mask shaped exactly like the matrix blocks will be placed on top of the micromodel picture. After these steps, pictures are ready to be processed. Using the image processing toolbox of MATLAB and two different sets of codes, masked pictures of the micromodel at different stages of the test will be analyzed and porosity, effective porosity and permeability will be calculated for each time step. In the following paragraphs, two sets of codes will be explained.

#### Porosity

**Fig. 3:**
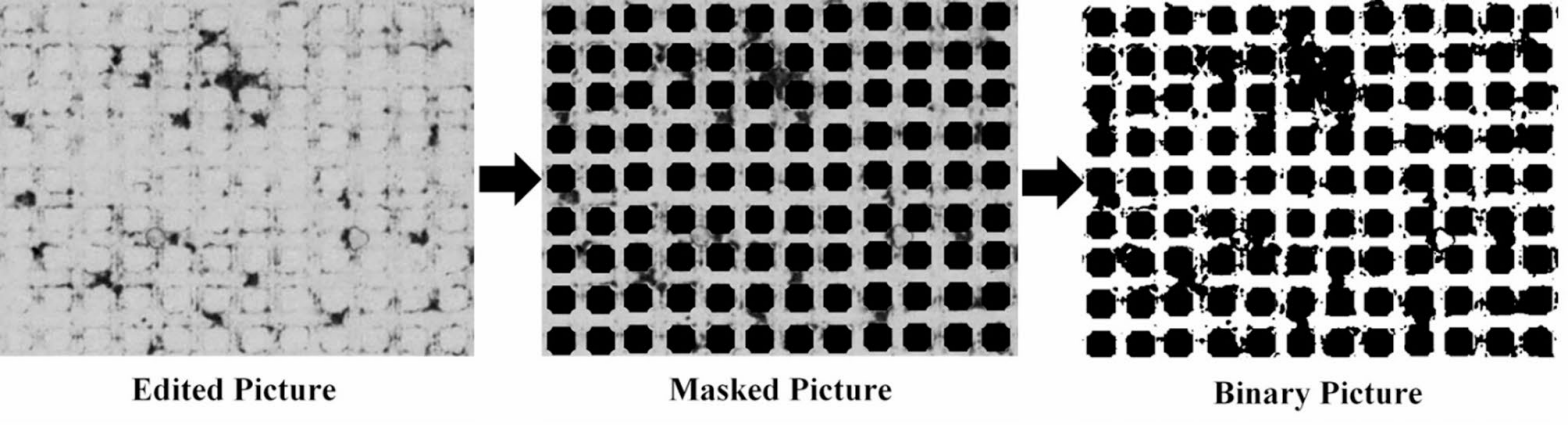
Masking and thresholding example; Edited picture was generated using Adobe Photoshop Lightroom Classic (v9.1; Adobe Inc.; https://www.adobe.com/products/photoshop-lightroom-classic.html); Binary picture was generated using MATLAB (vR2019b; The MathWorks Inc.; https://www.mathworks.com/products/matlab.html); Masked picture and entire image were generated using CorelDraw (v24.4.0.636; Corel Corporation; https://www.coreldraw.com/en/).

For porosity calculation, masked images were introduced to the code as a greyscale matrix. As the indication of brightness, each pixel was valued from 0 (completely black) to 255 (completely white). To distinguish the pore space from the matrix which consists of matrix block and clays, the greyscale matrix should be converted to a binary matrix. In a binary matrix, each pixel is valued 0 (black) or 1 (white). Conversion of the greyscale matrix to binary was done using the thresholding technique. In order to do so a threshold value was chosen by the user and every pixel of the greyscale matrix will be compared to it. If the greyscale value was greater than the threshold, pixel would be valued 1 and considered to be white, otherwise, it would be valued 0 and considered to be black. Procedures of masking and thresholding until reaching a binary image are shown in Fig. 3.

By counting the white (pore volume) and total number of pixels (bulk volume) and using the Eq. (1), porosity can be calculated.

1 $$\:\varnothing\:=\:\frac{Number\:of\:white\:pixels}{Total\:EquationNumber\:of\:pixels}\:\times\:100.\:\:\:\:\:\:\:\:\:\:\:\:\:\:\:\:\:\:\:\:\:\:$$

#### Effective porosity and permeability

**Fig. 4:**
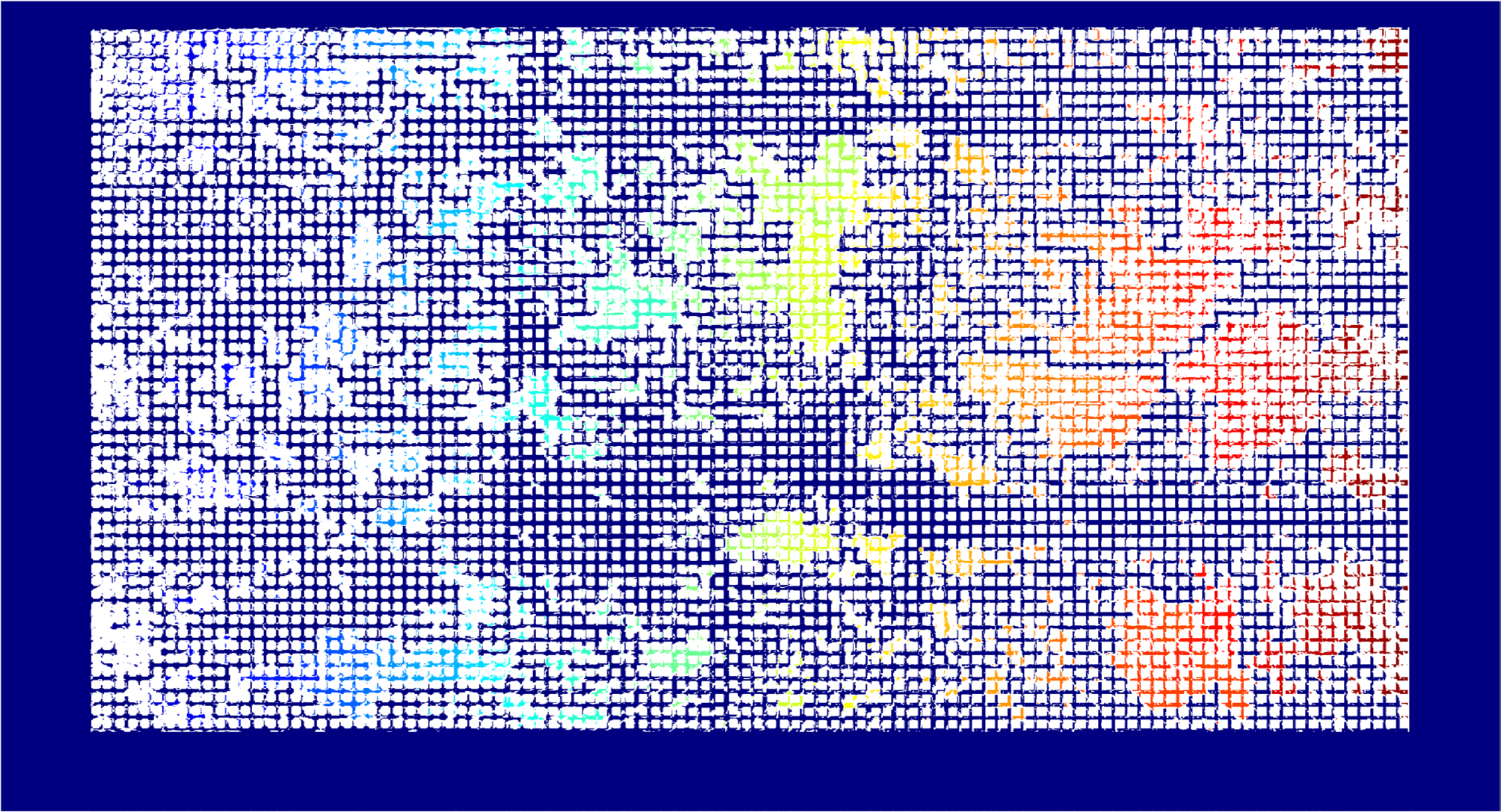
Labelled picture of micromodel’s porous media which connected and isolated pores can be distinguished generated using MATLAB (vR2019b; The MathWorks Inc.; https://www.mathworks.com/products/matlab.html).

The code that was explained before, calculates the total porosity of the micromodel which includes both connected and isolated pores. Effective porosity needs to be calculated as it’s the connected pores that contribute to the fluid flow and in other words determine the permeability and not the isolated ones. As the test continues, by the clay swelling and migration inside of the porous media of micromodel, connected pores can be isolated and isolated pores can open up and contribute to the flow, therefore effective porosity should be calculated at every time step to obtain a dynamic behavior of the permeability versus time. The idea behind our code was inspired by a previous study by Sharifipour et al. which used the connected component labeling technique^85^. In this study, we also used this technique to identify the connected pores. The code used the binary images and removed the isolated clusters from labeled connected pores which are available as flow paths from one side of the micromodel to the other side of it in order to calculate the effective porosity. Figure 4, presents the labeled picture of a test at its end (after 12 h injection of distilled water) where the isolated pores were clustered using a different color than blue which is the connected pore space contributing to the flow.

**Fig. 5:**
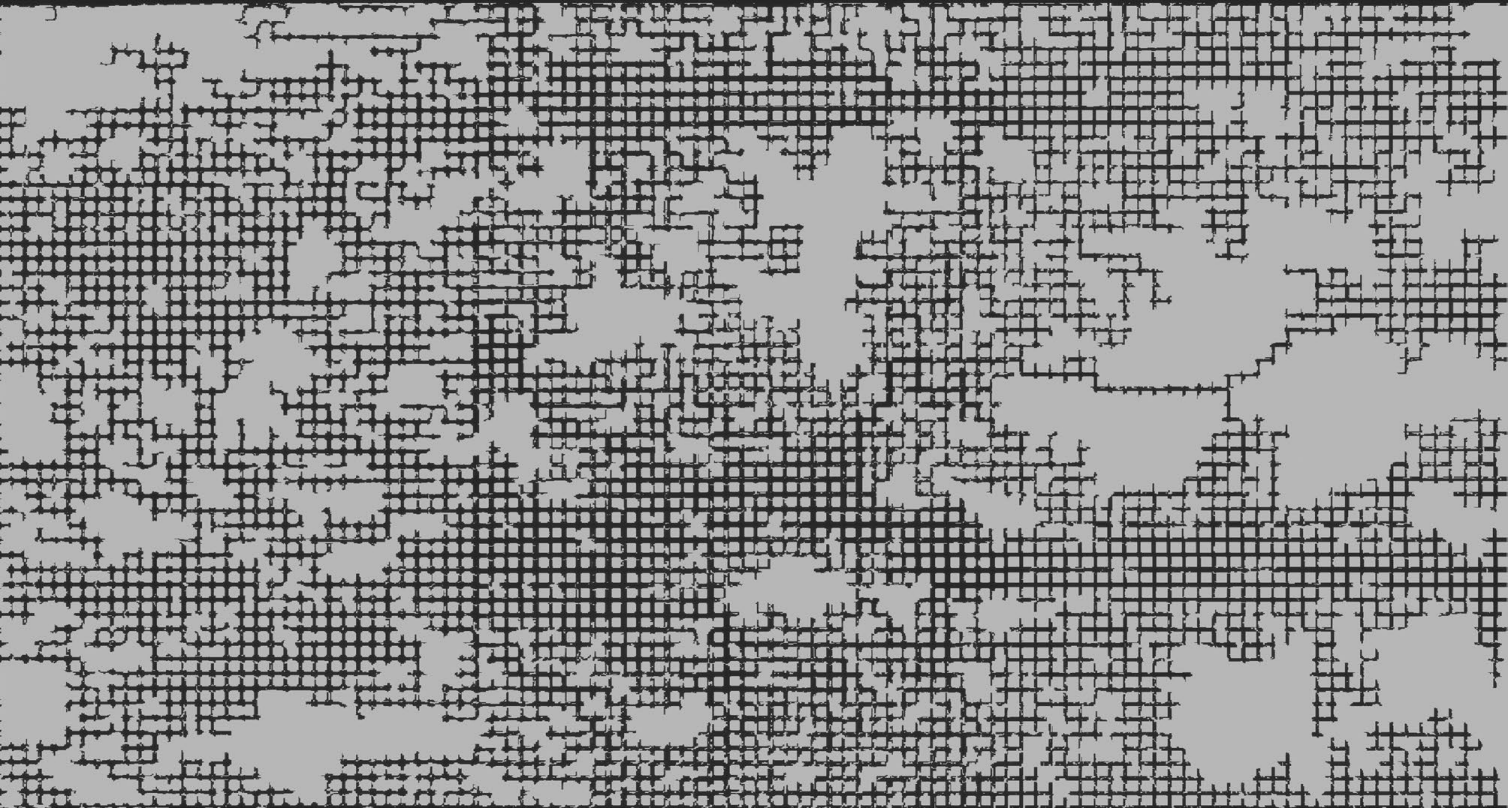
Isolated pores were eliminated and colored white as the matrix blocks and the pore space available for flow colored black; image was generated using MATLAB (vR2019b; The MathWorks Inc.; https://www.mathworks.com/products/matlab.html).

Now that the isolated pores have been recognized and labeled, they can be eliminated from the porous media which can be seen in Fig. 5. After reaching this point, similar to the porosity calculation by counting the black and white pixels, effective porosity will be calculated.

In order to calculate the permeability of the micromodel, the Kozney-Carman equation was used which is shown in Eq. (2)^86^.

2 $$\:K=C\frac{{\varnothing\:}^{3}}{{S}^{2}},\:\:\:\:\:\:\:\:\:$$

Where *K* is the permeability of the micromodel, *Ø* is the effective porosity, and *S* is the specific surface area and *C* is the Kozney factor that can be calculated using effective porosity and Eq. (3)^87^.

3 $$\:C=\frac{1}{\left(4\text{cos}\left(\frac{1}{3}\text{arccos}\left(2\varnothing\:-1\right)+\frac{4}{3}\pi\:\right)+4\right)}\:\:\:\:$$

The specific surface is the ratio of the inner surface of the rock to rock volume and for porous media with rectangle shaped cross-sectional pore geometries can be calculated using Eq. (4)^88^.

4 $$\:S=\frac{4\varnothing\:}{a}\:\:\:\:\left(4\right)$$

$$\:\:a\:\left(throat\:diameter\right)=100\:\mu\:m\:\:\:$$

## Results and discussion

According to the test procedure described in the previous section, 20 tests were conducted. Tests 1 to 5 were related to freshwater injection into the bentonite-coated micromodel which was saturated with five different formation waters at ambient temperature (25 °C). Tests No.6 to 10 were related to freshwater injection into the same bentonite-coated micromodel and saturated with the same five different formation waters. However, the testing temperature was raised to 70 °C. These 10 tests were designed to study the effect of temperature on the swelling and migration behavior of bentonite clay in combination with each other. Tests 11 to 20 were conducted with the same procedure but only in a kaolinite-coated micromodel instead of bentonite at 25 °C (Tests 11 to 15) and 70 °C (Tests 16 to 20) as testing temperatures. This set of tests was conducted to investigate the effect of temperature only on the migration behavior of clay such as kaolinite. During each test from its initiation and every hour of the test for 12 h., images were taken from the micromodel. Using image analysis, the porosity, effective porosity, and permeability of the clay-coated micromodel were calculated and their trend against their initial value were graphed versus the PV of the injected fresh water. Each of these parameters can give useful insights related to the damaging behavior of the clays such as swelling that can be seen in a downward shift in the porosity ratio graph due to increased volume of bentonite or an upward shift in the porosity chart can be due to clays exiting the porous media during migration. Also from the reduction of permeability as a result of effective porosity decline, clogging of throats due to clay swelling and migration can be concluded.

Results are presented here in the form of charts, in 2 sub-sections one for bentonite and another for kaolinite clay. Each figure contains two charts, (A): the porosity and effective porosity ratios of the clay-coated micromodel (porosity ratio against its initial value) versus LSW injected PV at 25 °C (solid lines) and 70 °C (dashed lines), and (B): The ratio of permeability of each time step to its initial value as permeability impairment versus the LSW injected PV.

### Bentonite

**Fig. 6:**
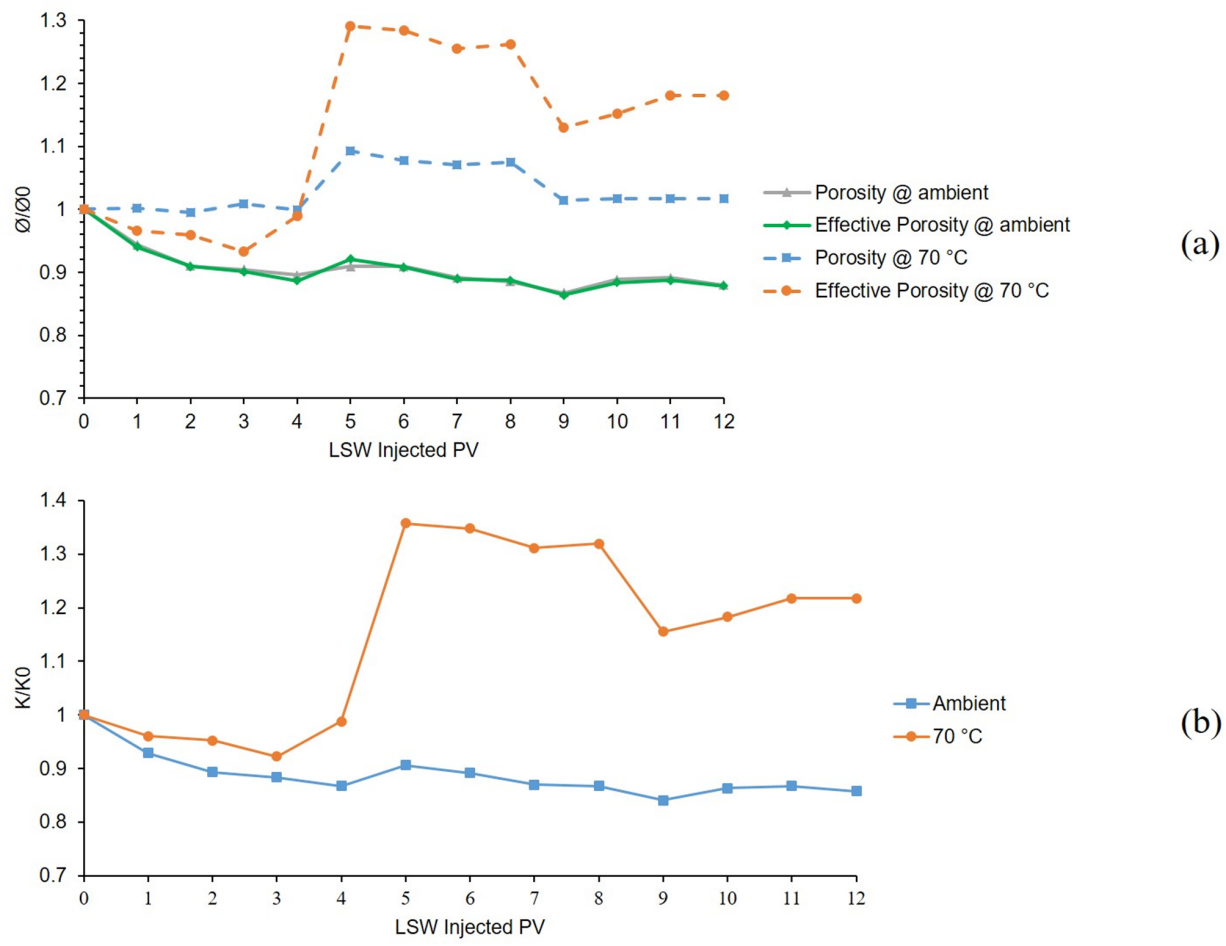
Results for 1st and 6th tests conducted in a bentonite-coated micromodel saturated with 60,000 ppm NaCl brine in 25 °C and 70 °C. (**a**) Porosity and effective porosity ratio vs. LSW injected PV for 1st and 6th tests. (**b**) Permeability impairment vs. LSW injected PV for 1st and 6th tests.

The first and 6th tests were conducted in a bentonite-coated micromodel which was saturated with 60,000 ppm NaCl brine as the formation water. The first test was conducted at 25 °C and 6th test at 70 °C. The results of these two tests are presented in Fig. 6. As it can be seen from the chart (A) of Fig. 6, the gray solid line shows the porosity’s downward shift in the first 4 PVs of LSW injection which is due to swelling of bentonite. After that until 6 PV, a slightly upward shift can be seen in porosity which is related to initiation of bentonite detachment from the surface of the pore walls and migrating out of the micromodel. Bentonite swelling and porosity reduction continued until 9 PV and after that, till the end of the test, it seems that swelling was stopped and migration was still taking place. The effective porosity line at ambient temperature (solid green line) follows the same trend as the porosity. It seems that in the temperature of 70 °C, the porosity (dashed blue line) in the first 4 h (4 PV) of LSW injection remained constant but the effective porosity (dashed orange line) shows downward and upward fluctuations at the same time. The reason behind these findings is that the decreasing and increasing effects in the porosity value due to the bentonite swelling and migration out of the micromodel neutralized each other which maintained the porosity value constant. After that, both porosity and effective porosity values were increased due to the migration of bentonite clays out of the micromodel having a stronger effect than swelling on the porosity values. As time goes on, both charts show decreasing trends, especially between 8 and 9 h of LSW injection which shows the end of the migration at 8 and the end of swelling in 9 h of LSW injection.

The permeability chart is shown in Fig. 6. B, the blue line shows the declining trend from the initial permeability of the micromodel for the 1st test which was conducted at ambient temperature due to clay swelling which lowers the permeability of the micromodel as low as 85% of its initial value. But as for the 6th test which was conducted at 70 °C, due to the escalated migration at this temperature and clays exiting the porous media, permeability increased as high as 1.35 times the initial value and again lowered due to the swelling.

These two tests were conducted in the presence of Na^+^ cation, which is not able to control the clay swelling and migration, and results showed that at elevated temperature, migration would be escalated and force the clays out of the micromodel that is shown in the increasing values of porosity, effective porosity and permeability.

**Fig. 7:**
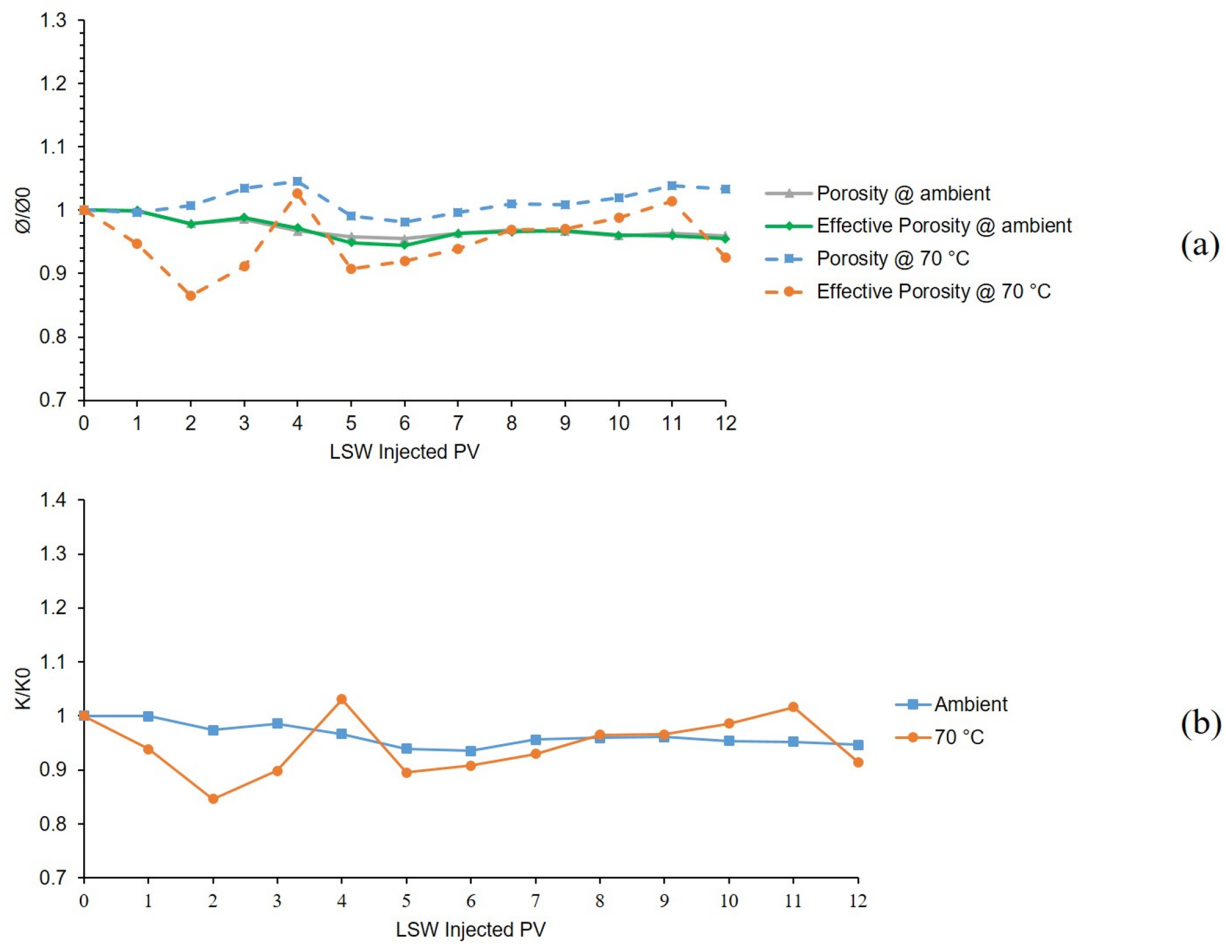
Results for 2nd and 7th tests conducted in a bentonite-coated micromodel saturated with 60,000 ppm KCl brine in 25 °C and 70 °C. (**a**) Porosity and effective porosity ratio vs. LSW injected PV for 2nd and 7th tests. (**b**) Permeability impairment vs. LSW injected PV for 2nd and 7th tests.

The 2nd and 7th tests were also conducted in a bentonite-coated micromodel saturated with 60,000 KCl brine as the formation water. Second test was conducted at 25 °C and 7th test at 70 °C. Figure 7a, shows the porosity and effective porosity ratios for these two tests during 12 h of LSW injection and Fig. 7b, presents the permeability chart for the mentioned tests. As can be seen from Fig. 7a, porosity and effective porosity ratio for 2nd test (ambient temperature) are generally declining due to the clay swelling and with some minor increase due to migration. But in the case of the 7th test, due to the escalated migration at elevated temperatures, the chart shows a declining and noticeably increasing trend. Figure 7b, shows the permeability values related to these two tests. The ambient chart shows a declining trend due to the swelling but the 70 °C chart shows declining and increasing fluctuations due to both swelling and escalated migration at elevated temperature.

By comparing the results of 2nd and 7th to 1st and 6th tests, the effectiveness of K^+^ cation in the case of controlling swelling and migration of bentonite clay is evident compared to Na^+^. Although even by the K^+^’s ability to mitigate formation damage, the effect of temperature in magnifying clay migration is clear.

**Fig. 8:**
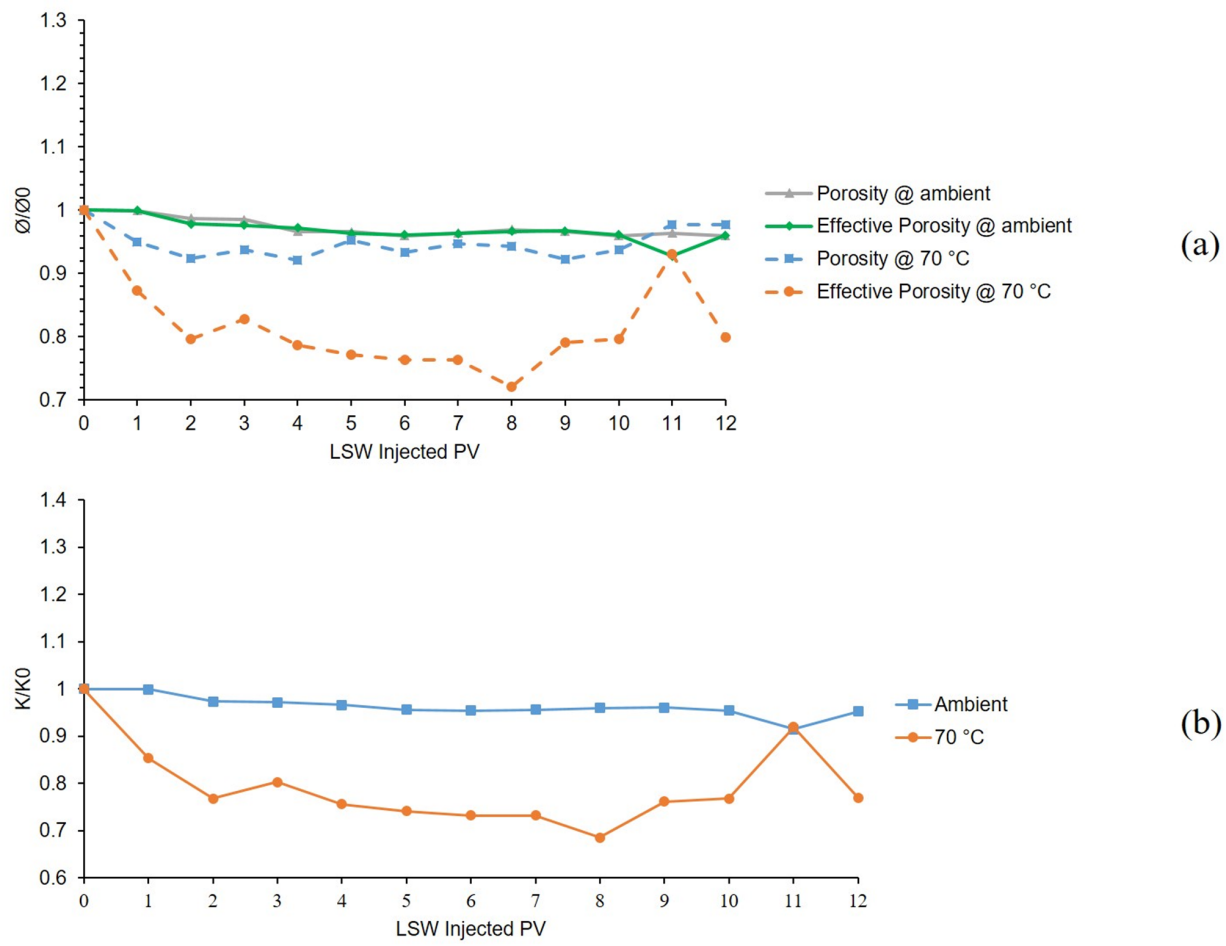
Results for 3rd and 8th tests conducted in a bentonite-coated micromodel saturated with 60,000 ppm CaCl_2_ brine in 25 °C and 70 °C. (**a**) Porosity and effective porosity ratio vs. LSW injected PV for 3rd and 8th tests. (**b**) Permeability impairment vs. LSW injected PV for 3rd and 8th tests.

The 3rd and 8th tests were conducted at temperatures of 25 °C and 70 °C in a bentonite-coated micromodel saturated with 60,000 ppm CaCl_2_ brine. Results of 12 h LSW injection into the mentioned micromodel are presented in Fig. 8. From the porosity and effective porosity ratio charts for the 3rd test conducted at 25 °C which are shown as grey and green solid lines in Fig. 8. A, clay swelling can be seen from declining trends of the charts in the early hours of LSW injection. After 4 h, the values seem to be stabilized which indicates efficient control of clay swelling and migration. However, this is not the case for the 8th test which was conducted at 70 °C (dashed blue and orange lines in Fig. 8A). Same as before, clay swelling is evident at early hours but some fluctuations in the porosity and effective porosity, suggest magnified clay migration compared to the 3rd test at ambient temperature.

From Fig. 8B which presents permeability results for these two tests, it can be said that clay swelling was in action which can be seen from the declining trends of both ambient and 70 °C even more severe than the tests using K^+^. Clay migration was controlled noticeably by Ca^2+^, as fewer fluctuations can be seen from both tests compared to Na^+^ and even K^+^. But also with this level of control, increased temperature resulted in escalated migration which can be concluded from a higher level of formation damage and some fluctuation in the permeability of 70 °C (orange line) compared to ambient (blue line).

**Fig. 9:**
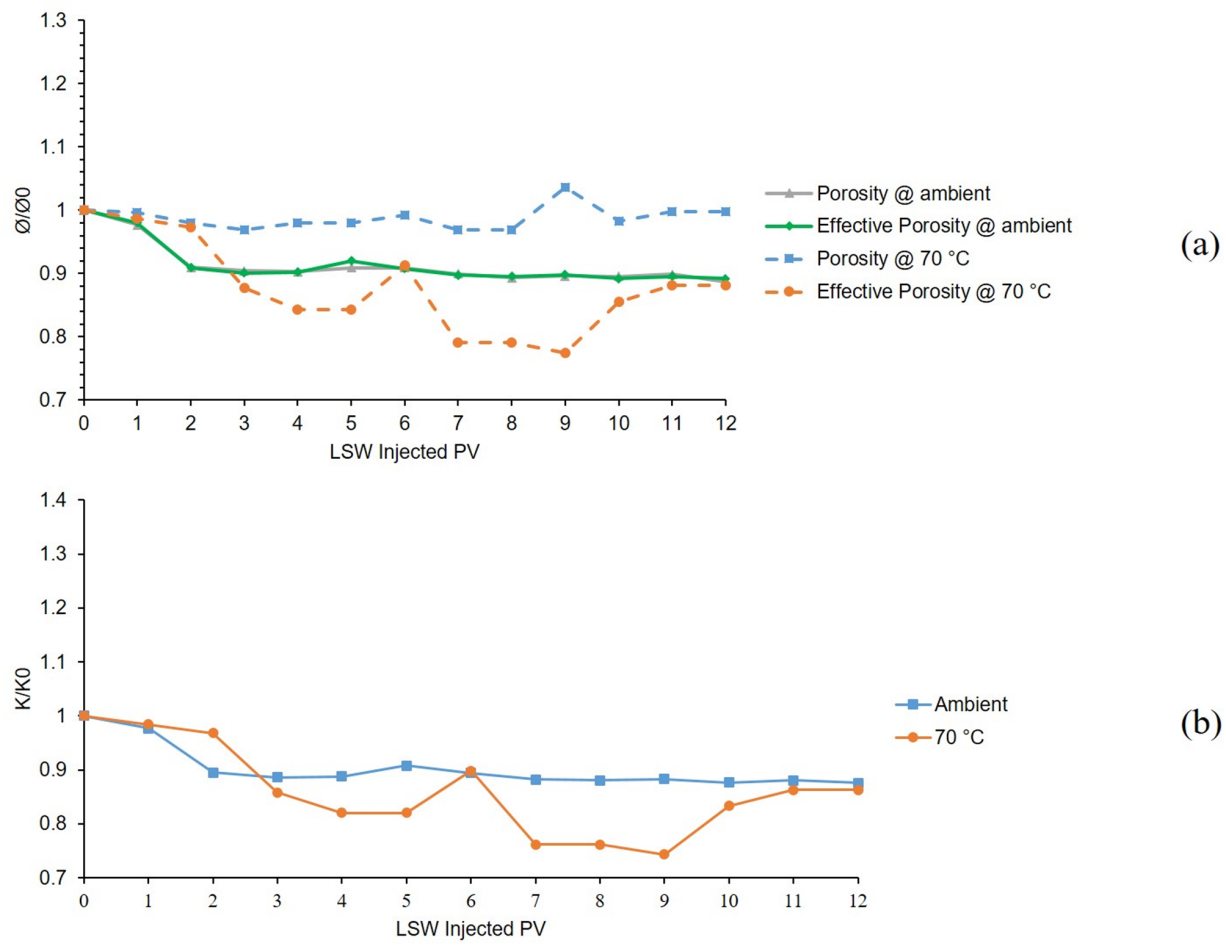
Results for 4th and 9th tests conducted in a bentonite-coated micromodel saturated with 60,000 ppm NaCl + CaCl_2_ brine in 25 °C and 70 °C. (**a**) Porosity and effective porosity ratio vs. LSW injected PV for 4th and 9th tests. (**b**) Permeability impairment vs. LSW injected PV for 4th and 9th tests.

Figure 9, presents the results of 4th and 9th tests, which were conducted in a bentonite-coated micromodel saturated with 60,000 ppm NaCl + CaCl_2_ as formation water. In the 4th test, in which LSW was injected into the micromodel at 25 °C for 12 h, clay swelling was the main formation damage mechanism seen from the declining trend in porosity and effective porosity ratio in dashed grey and green lines in Fig. 9A. Due to the presence of Ca^2+^, clay migration was controlled considerably. But in the case of the 9th test which was conducted at 70 °C, besides swelling, migration of clays and them exiting the micromodel is evident from the porosity trend. As can be seen from the dashed blue line of Fig. 9A, in the early hours of the LSW injection at 70 °C, the declining effect of swelling on porosity was neutralized by the incremental effect of clays migrating out of the micromodel and made the porosity value constant over time. By comparing the results of 6th and 9th tests, it can be concluded that by adding Ca^2+^ to Na^+^ cation in the formation water, clay migration was considerably mitigated as fewer fluctuations can be seen from the 9th test chart. Permeability trends for these two tests are presented in Fig. 9B, supports the findings from porosity charts.

**Fig. 10:**
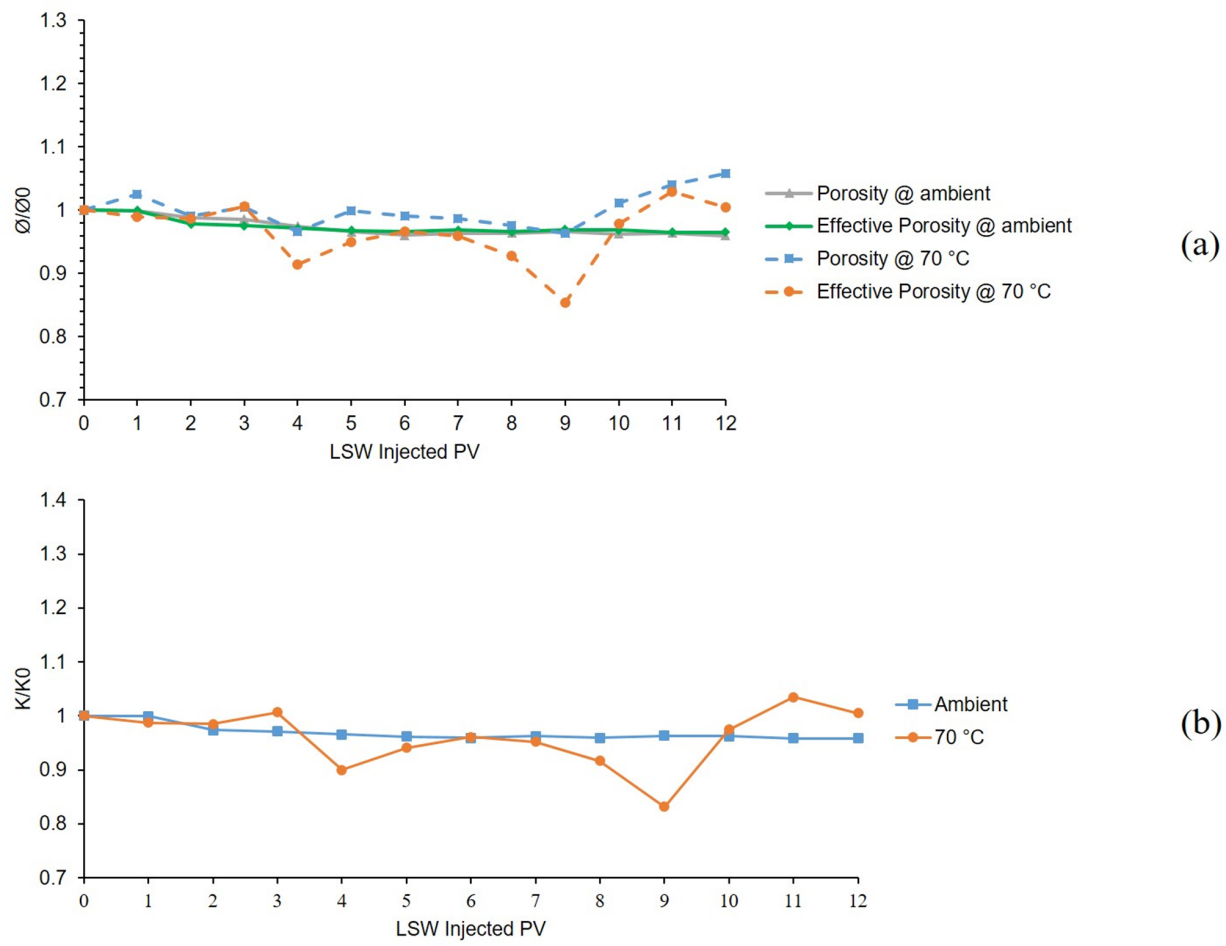
Results for 5th and 10th tests conducted in a bentonite-coated micromodel saturated with 60,000 ppm KCl + CaCl_2_ brine in 25 °C and 70 °C. (**a**) Porosity and effective porosity ratio vs. LSW injected PV for 5th and 10th tests. (**b**) Permeability impairment vs. LSW injected PV for 5th and 10th tests.

Figure 10, presents the results of the 5th and 10th tests, which were conducted in a bentonite-coated micromodel saturated with 60,000 ppm KCl + CaCl_2_ brine. Porosity ratio results of the 5th test which can be seen as solid grey and green lines in Fig. 10A, show great control of clay swelling and migration as the porosity trends display minimal changes at 25 °C. On the other hand, by raising the temperature to 70 °C in the 10th test, porosity trends seem to fluctuate due to escalated clay migration. Generally, charts display increasing trends, which resulted from the controlled swelling due to the presence of K^+^ and Ca^2+^ cations, and escalated migration of clays out of the micromodel due to the higher temperature. Figure 10B, presents the permeability results for these two tests, which show the great control of damage in 25 °C and fluctuated permeability trend for 70 °C due to escalated clay migration.

### Kaolinite

**Fig. 11:**
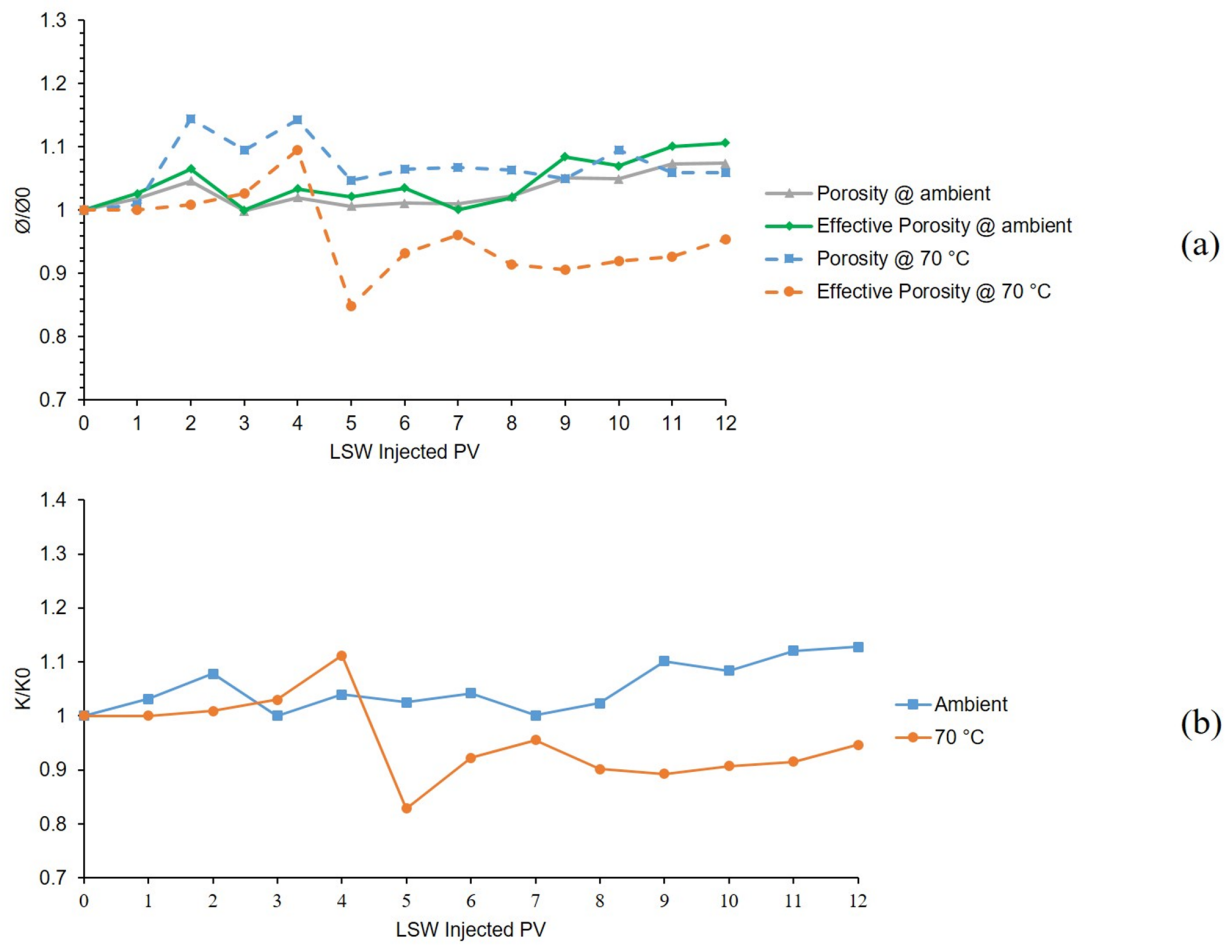
Results for 11th and 16th tests conducted in a kaolinite-coated micromodel saturated with 60,000 ppm NaCl brine in 25 °C and 70 °C. (**a**) Porosity and effective porosity ratio vs. LSW injected PV for 11th and 16th tests. (**b**) Permeability impairment vs. LSW injected PV for 11th and 16th tests.

The 11th and 16th tests were conducted in a kaolinite-coated micromodel saturated with 60,000 ppm NaCl brine as formation water at 25 °C and 70 °C. The results of these tests are presented in Fig. 11. As can be seen from the solid grey and green solid lines in Fig. 11A, porosity and effective porosity ratio values for the test conducted at 25 °C, show general increasing trends due to migration of kaolinite clays out of the micromodel. The dashed blue line in Fig. 11A, presents the porosity ratio changes during 12 h of LSW injection at 70 °C. During the first 4 h of injection, as clays migrate toward out of the micromodel, an incremental trend was seen. However, after that, the porosity value decreased and until the end of the test, displayed a constant trend. The reason behind this decrease in calculated porosity value lies in the 2D nature of the image processing technique. In other words, as the temperature was elevated to 70 °C, clay migration was intensified and forced the kaolinite particles to clog the throats to form bigger and darker black spots in the image and force the kaolinite particles that were stacked on top of each other in depth of the porous media to be released and display a larger number of black pixels. This would partially increase the number of black pixels seen and processed by the image processing code which showed itself as a decrease in porosity value. The effective porosity ratio trend shown as dashed orange line in Fig. 11A, supports this notion, as effective porosity was first increased and then decreased as more throats were clogged and more pore space was isolated from the flow path.

Figure 11B, presents the permeability charts with these two tests and as it is evident, permeability for the 11th test which was conducted at ambient temperature was generally increased as the migration of kaolinite particles was not that severe which resulted in them having the opportunity to migrate toward out of the micromodel. However, by raising the temperature to 70 °C, migration was so intensified that kaolinite particles clogged the porous media’s throats in their migration path which displayed a decreasing trend for the permeability.

**Fig. 12:**
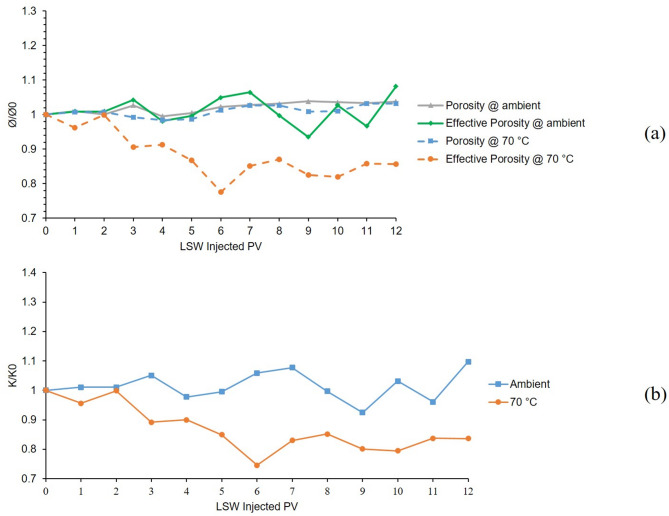
Results for 12th and 17th tests conducted in a kaolinite-coated micromodel saturated with 60,000 ppm KCl brine in 25 °C and 70 °C. (**a**) Porosity and effective porosity ratio vs. LSW injected PV for 12th and 17th tests. (**b**) Permeability impairment vs. LSW injected PV for 12th and 17th tests.

Figure 12 presents the results of 12th and 17th test, which were conducted in a kaolinite-coated micromodel saturated with 60,000 ppm KCl brine at 25 °C and 70 °C. As can be seen from the porosity results of the 12th test (solid grey line in Fig. 12A) which was conducted at 25 °C, the slightly increasing trend due to kaolinite migration out of the micromodel is evident. The effective porosity ratio trend for this test (solid green line in Fig. 12A) however fluctuated for the same reason as pore spaces will continuously be isolated and again opened to fluid flow. The porosity ratio chart for the 17th test (dashed blue line in Fig. 12A) which was conducted at 70 °C, follows the same increasing trend as the 12th test but the effective porosity chart shows a great decrease for the 17th test due to elevated temperature effect on escalating migration, that resulted in clogging throats and reduction of available pore space to the fluid flow. Figure 12B which displays permeability results for these two tests, supports the mentioned events as permeability for ambient temperature shows a generally increasing trend and decreasing trend for 70 °C. By the results obtained from these two tests, it seems that K^+^ cation although able to control migration in ambient temperature, wasn’t capable of controlling clay migration at elevated temperature.

**Fig. 13:**
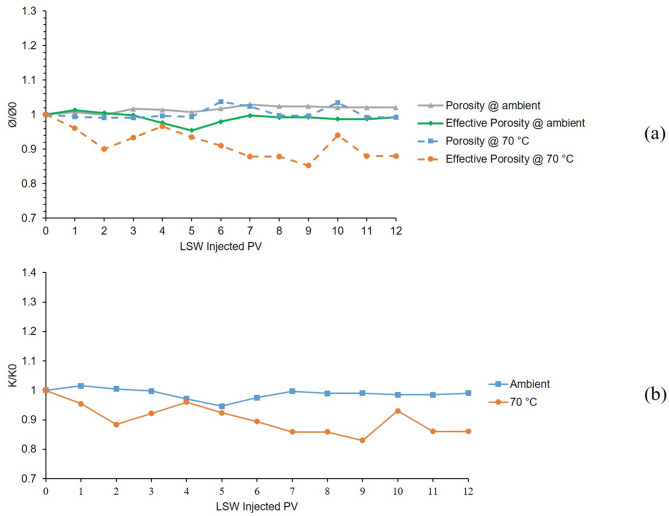
Results for 13th and 18th tests conducted in a kaolinite-coated micromodel saturated with 60,000 ppm CaCl_2_ brine in 25 °C and 70 °C. (**a**) Porosity and effective porosity ratio vs. LSW injected PV for 13th and 18th tests. (**b**) Permeability impairment vs. LSW injected PV for 13th and 18th tests.

Results of 13th and 18th tests which were conducted at 25 °C and 70 °C, in a kaolinite-coated micromodel saturated with 60,000 ppm CaCl_2_ brine as formation water are presented in Fig. 13. As can be seen from the solid grey and green lines of Fig. 13A, related to porosity and effective porosity ratio values at 25 °C, the great control of kaolinite migration by the Ca^2+^ cation at this temperature is evident. But in the case of 18th test which was conducted at 70 °C, intensified kaolinite migration can be seen from fluctuated trends in the dashed blue and orange lines. However, by comparing the effective porosity ratio of the tests conducted at 25 °C and 70 °C in the presence of Na^+^, K^+^ and Ca^2+^ cations, it can be concluded that Ca^2+^ displayed a better ability to control clay migration compared to the other cations even in elevated temperature. Permeability results of the 13th and 18th that are presented in Fig. 13B, support the mentioned findings. The permeability remained fairly constant throughout the test conducted at ambient temperature and less damaged in 70 °C.

**Fig. 14:**
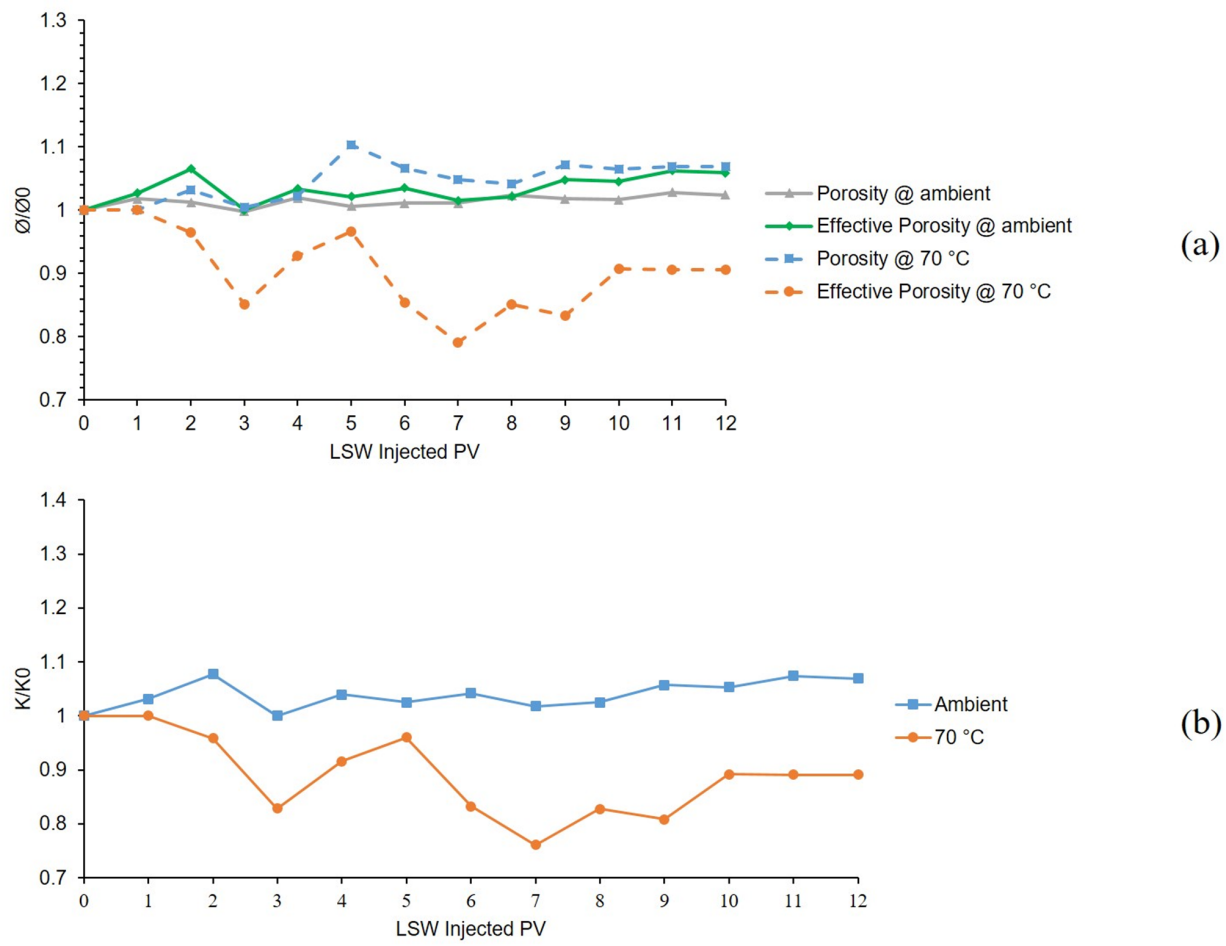
Results for 14th and 19th tests conducted in a kaolinite-coated micromodel saturated with 60,000 ppm NaCl + CaCl_2_ brine in 25 °C and 70 °C. (**a**) Porosity and effective porosity ratio vs. LSW injected PV for 14th and 19th tests. (**b**) Permeability impairment vs. LSW injected PV for 14th and 19th tests.

Figure 14 presents the results for the 14th and 19th tests conducted in kaolinite-coated micromodel saturated with 60,000 ppm NaCl + CaCl_2_ brine in two different temperatures of 25 and 70 °C. During 14th test at 25 °C, porosity and effective porosity ratio (solid grey and green lines in Fig. 14A) seem fairly constant due to the control of migration by the presence of the Ca^2+^ cation alongside Na^+^. But during the 19th test as temperature increased to 70 °C, porosity and effective porosity ratio charts (dashed blue and orange lines in Fig. 14A), displayed similar behavior as 16th test due to the sodium cation’s lack of ability to control clay migration. Permeability impairment trend presented in Fig. 14B, shows the kaolinites migrating out of the micromodel at ambient temperature and clogging throats at 70 °C as clay migration escalated at this temperature.

**Fig. 15:**
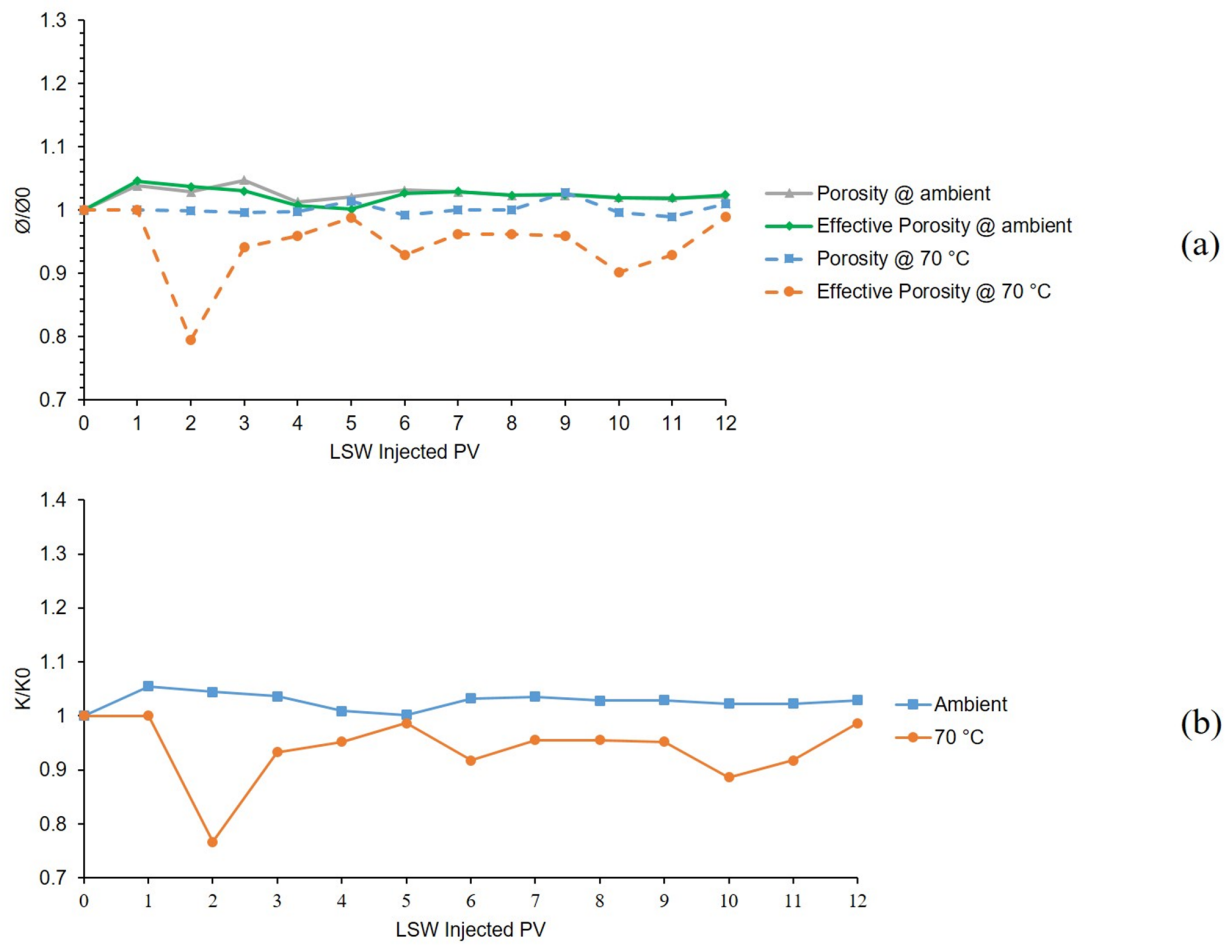
Results for 15th and 20th tests conducted in a kaolinite-coated micromodel saturated with 60,000 ppm KCl + CaCl_2_ brine in 25 °C and 70 °C. (**a**) Porosity and effective porosity ratio vs. LSW injected PV for 15th and 20th tests. (**b**) Permeability impairment vs. LSW injected PV for 15th and 20th tests.

The 15th and 20th tests were conducted at 25 °C and 70 °C in a kaolinite-coated micromodel saturated with 60,000 ppm KCl + CaCl_2_ brine as formation water. It seems from, solid grey and green lines in Fig. 15A, that porosity and effective porosity ratios were fairly maintained constant during the test conducted at 25 °C due to great control of clay migration at this temperature. However, raising the temperature to 70 °C, made clay migration more severe which can be seen from fluctuated trends of porosity and effective porosity ratio related to the 20th test (dashed blue and orange lines in Fig. 15A). By taking a look at the permeability impairment chart displayed in Fig. 15B, the clay migration out of the micromodel can be seen from the test conducted at ambient temperature and clogging throats due to escalated migration is evident from the test conducted at 70 °C. Results of these two tests and comparing them to the 13th and 18th (CaCl_2_ formation water) also support the claim that K^+^ isn’t as capable as Ca^2+^ cation in controlling clay migration especially at elevated temperature.

All of the presented results were extracted from micromodel images using an image processing technique. The mentioned claims regarding the events that took place in the porous media of the micromodel (clay swelling, throat clogging, and clays exiting the micromodel due to clay migration) were in line with the visual evidence observed from the taken images.

## Conclusions

In this study, a total number of 20 tests were designed and conducted in glass micromodels coated with bentonite and kaolinite clays which were saturated with 5 different formation waters. During tests, fresh water was injected into the micromodels for 12 h in two different temperatures (25 °C and 70 °C) to investigate the effect of temperature and cation type on the severity of induced clay swelling and migration. Images were taken from the micromodel every hour and were processed to extract porosity, effective porosity and permeability impairment during the test. Major conclusions from this study are presented below:

- Increasing temperature didn’t magnify the swelling of bentonite clay.
- Clay migration for both kaolinite and bentonite was escalated by increasing temperature.
- Kaolinite migration increased more with rising temperature compared to bentonite. The kaolinite particles were so numerous that they clogged the pore throats, unlike bentonite, which tended to exit the micromodel.
- Clay swelling was better controlled by K^+^ cation compared to Ca^2+^ which is related to K^+^ smaller radius and higher charge density.
- Both K^+^ and Ca^2+^ cations showed equally great potential in controlling clay migration at ambient temperature. However, at elevated temperature (70 °C), Ca^2+^ showed better performance in controlling migration.
- Clay migration at higher temperature was so severe that even Ca^2+^ cation that controlled the clay migration at ambient temperature hadn’t been able to fully control it.

## Data Availability

The authors declare that the data supporting the findings of this study are available within the paper. Should any raw data files be needed in another format they are available from the corresponding author upon reasonable request.

## Glossary

Low salinity water (LSW): Water that has a salt concentration about one-tenth that of formation water.
Low salinity waterflooding (LSWF): Low salinity waterflooding is an oil recovery method that injects low-salinity water to improve oil recovery.
Critical salt concentration (CSC): Salinity threshold below which clay particles in the reservoir become unstable, potentially leading to swelling, migration, and formation damage.
Cation exchange capacity (CEC): Measure of clay’s ability to attract and hold cations, influencing fluid-rock interactions in reservoir conditions.
The Derjaguin–Landau–Verwey–Overbeek theory (DLVO): Explains the stability of colloidal particles by balancing attractive van der Waals forces and repulsive electrostatic forces between them.
Pore volume (PV): Total volume of the void spaces within the porous media that can store fluids like oil, water, or gas.
